# IFT20 regulates TFEB-dependent lytic granule biogenesis in cytotoxic T lymphocytes by orchestrating the MPR-dependent transport of granzyme B

**DOI:** 10.1038/s41419-025-07727-5

**Published:** 2025-05-19

**Authors:** Francesca Finetti, Fabrizia Zevolini, Loredana Migliore, Valentina Cianfanelli, Leandro Marzuoli, Nagaja Capitani, Chiara Cassioli, Laura Patrussi, Cristina Ulivieri, Giuseppe Marotta, Cosima T. Baldari

**Affiliations:** 1 Department of Life Sciences, University of Siena, Siena, Italy; 2 Department of Science, University “ROMA TRE”, Rome, Italy; 3 Department of Woman and Child Health and Public Health, Gynecologic Oncology Unit, Fondazione Policlinico Universitario A. Gemelli IRCCS, Rome, Italy; 4 Siena University Hospital, Siena, Italy

**Keywords:** Immunology, Cell biology

## Abstract

Cytotoxic T lymphocytes (CTL) exploit specialized secretory lysosomes, the lytic granules (LG) to kill target cells. The LGs carry a battery of apoptosis-inducing molecules enriched in granzymes (GZM), perforin and FasL, which are released at the immune synapse formed by CTLs with their cognate targets. Recent studies have revealed an unexpected diversity among LGs, suggesting the existence of multiple vesicular trafficking pathways in their biogenesis and exocytosis. We have previously implicated the ciliary protein IFT20 in the retrograde trafficking of the cation-independent mannose-6-phosphate receptor (MPR), which is required for the lysosomal targeting of the acid hydrolases. Here we investigate the role of IFT20 in LG biogenesis in CTLs, showing that IFT20 is essential for MPR recycling to the trans-Golgi network and ensures proper granzyme B (GZMB) localization to LGs. As a result, IFT20 deficiency impairs the killing capability of CTLs. In turn, to rescue the lysosome and LG defects, IFT20-deficient CTLs expresses higher levels of lysosomal genes and of components of the cytotoxic machinery of LGs. Interestingly, an in silico analysis suggests a transcriptional co-regulation of lysosome and LG genes by the master regulator of lysosome biogenesis TFEB. Accordingly, modulation of TFEB results in alterations in the expression of LG-related genes and CTL-mediated cytotoxicity. Collectively, our results identify IFT20 as a new player in the trafficking pathways that regulate LG biogenesis and highlight the existence in CTLs of an extended gene expression program regulated by TFEB, downstream of IFT20.

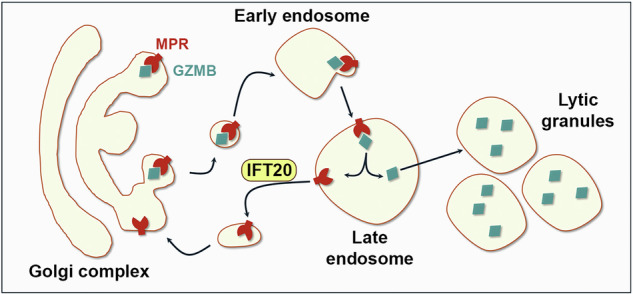

## Introduction

As key players in one of the fundamental mechanisms of immune response, lytic granules (LG) are a cornerstone of lymphocyte-mediated cytotoxicity, enabling cytotoxic T lymphocytes (CTL) and NK cells to eliminate virally infected and tumor cells through the exocytosis of lytic molecules. LGs are lysosome-related organelles (LRO) characterized by typical lysosomal markers, such as the lysosomal membrane protein LAMP1 and the hydrolases cathepsins (CTS) B and D, and containing in their lumen a set of cytolytic effectors, among which the pore-forming protein perforin (PRF) and the serine proteases granzymes (GZM), that are packed on a scaffold of the proteoglycan serglycin (SRGN) [1]. Upon CTL activation by major histocompatibility complex class I (MHCI)-bound antigen and assembly of the immunological synapse (IS), LGs undergo exocytosis at the synaptic membrane, thus selectively releasing their luminal contents onto the target cell [2]. The change in pH and [Ca^2+^] in the extracellular milieu allows the solubilization of PRF and GZMs and the activation of PRF, which oligomerizes and forms pores allowing GZMs to enter target cells and induce their apoptotic demise [3, 4]. Once killing of the engaged cell has been triggered, this process can be repeated on new cognate targets, making CTLs powerful serial killers [5].

The importance of LGs in the CTL-mediated anti-tumoral and anti-viral defenses is underscored by severe immune defects in human primary immunodeficiencies associated with abnormal CTL function. Identifying the gene mutations in these patients has established both LG components (e.g. PRF), and regulators of their biogenesis (e.g. AP3B1) and exocytosis (e.g. Munc 13-4, Munc 18-2, syntaxin 11, Rab27A, LYST), as causal to these diseases, and laid the foundation for mechanistic studies that have provided crucial insights into the vesicular trafficking pathways that regulate these processes [6–8]. However, the precise sequence of trafficking events coordinating LG biogenesis and release and the respective regulators remain to be fully elucidated.

The cation-independent mannose-6-phosphate (M6P) receptor (MPR) pathway is responsible for the lysosomal targeting of the acid hydrolases in all cell types [9]. Lysosomal hydrolases undergo a number of glycosylation events first in the endoplasmic reticulum and then in the cis-Golgi compartment. At the trans-Golgi network (TGN) they are tagged with a M6P moiety that mediates their binding to the MPR. MPRs bound to their M6P-tagged cargoes bud from the TGN as clathrin-coated vesicles and transit through the early endosomes before reaching the late endosomes. The progressive drop in pH promotes dissociation of the cargoes, which enter the lysosomes through fluid-phase endocytosis, while the MPRs are recycled back to the TGN by retrograde trafficking to pick up new cargoes [10]. Defects in this process lead to the formation of dysfunctional lysosomes, triggering a compensatory upregulation in the lysosome biogenesis gene program, known as the CLEAR network, coordinated by the master transcription factor TFEB [11]. Consistent with the lysosomal origin of LGs, the MPR pathway is also exploited by CTLs for the LG transport of the GZMs [12].

We have previously reported that the ciliogenesis protein IFT20 is essential for the retrograde trafficking of the MPR to the TGN through coupling the MPR to the (-) end microtubule motor dynein. IFT20 deficiency results in defective targeting of acid proteases and lipases to lysosomes and the generation of dysfunctional lysosomes both in lymphocytes and in non-immune cells, highlighting its key role in lysosome biogenesis [13]. We hypothesized that IFT20 may regulate the transport of the GZMs to LGs by promoting the retrograde traffic of the MPR to the TGN in CTLs. We show that in IFT20-deficient CTLs the MPR-dependent localization of GZMB to LGs is impaired, leading to dysfunctional LGs and defective target cell killing. Additionally, we show that TFEB coordinates not only the expression of the CLEAR gene network regulating lysosome biogenesis, but also the expression of the genes encoding LG components, providing evidence for an extended TFEB-regulated CLEAR network specific for CTLs.

## Results

### IFT20 is required for the retrograde transport of the MPR to the TGN and GZMB targeting to LGs in CTLs

**Fig. 1:**
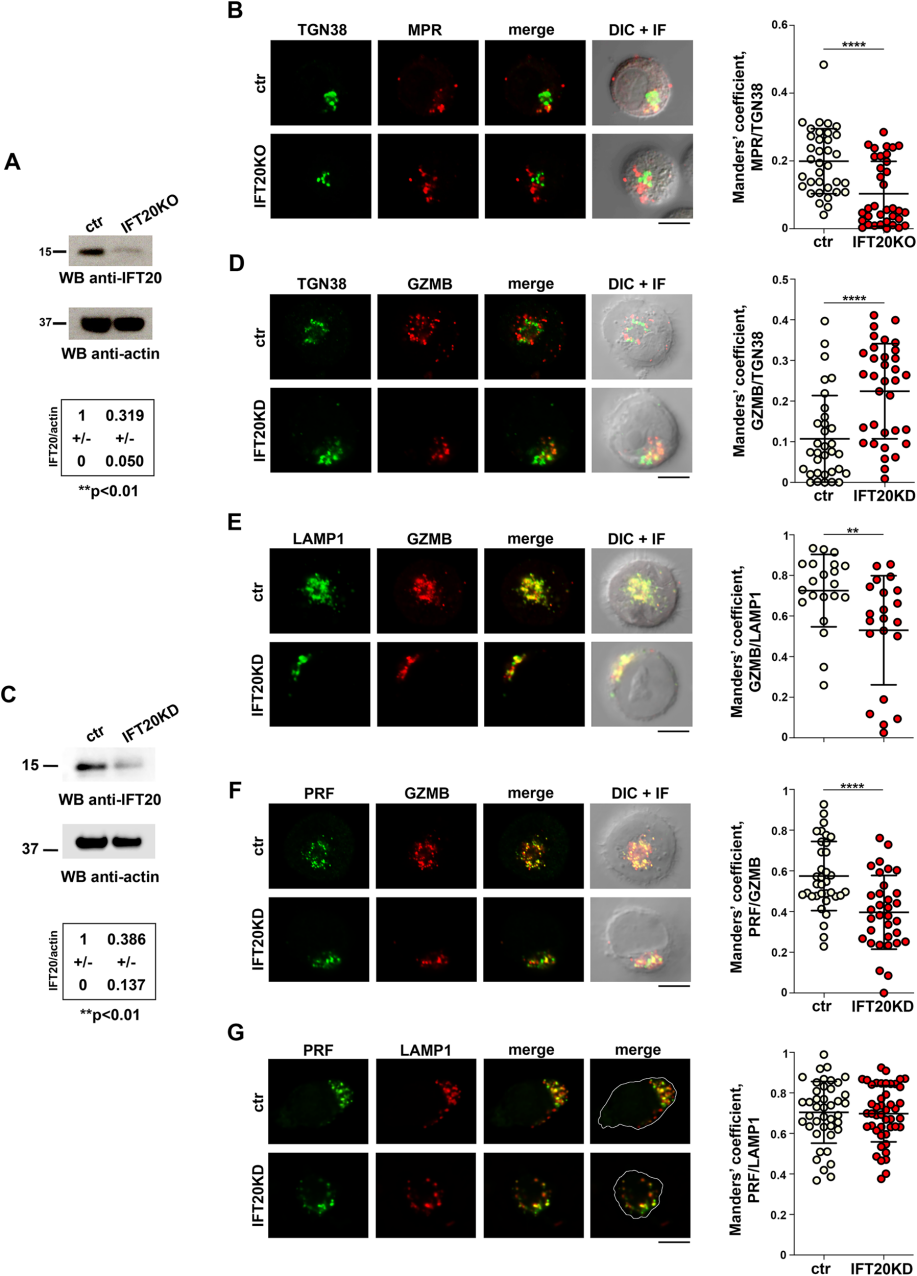
IFT20 controls MPR-mediated GZMB targeting to LGs. Immunoblot analysis of IFT20 in representative matched control (ctr) and IFT20 KO (**A**) and IFT20 KD (**C**) CTLs with respective loading control (actin). The migration of molecular mass markers is indicated. The quantification of IFT20 in CTL lysates is reported in the table (n = 3; mean fold ± SD, one-sample *t* test). Immunofluorescence analysis of recycled MPR and TGN38 (**B**) or GZMB-mCherry and TGN38 (**D**) or LAMP1 (**E**) in IFT20 KO (**B**) or IFT20 KD (**D**, **E**) CTLs. The graphs show the quantification using Manders’ coefficient of the weighted colocalization of recycled MPR and TGN38 (**B**) or GZMB-mCherry and TGN38 (**D**) or LAMP1 (**E**) in either ctr or IFT20-deficient CTLs. Immunofluorescence analysis of PRF and GZMB-mCherry (**F**) or LAMP1 (**G**) in ctr and IFT20 KD CTLs. The graphs show the quantification using Manders’ coefficient of the weighted colocalization of GZMB-mCherry and PRF (**F**) or PRF and LAMP1 (**G**). (≥35 cells from 3 independent experiments; mean fold ± SD, unpaired *t* test). Medial optical sections of representative images are shown. Scale bars: 5 μm. *P < 0.05; **P < 0.01; ***P < 0.001; ****P < 0.0001.

To investigate whether the lysosome biogenesis-related function of IFT20, involving retrograde trafficking of the MPR [13], is exploited for LG biogenesis, we measured MPR recycling in human CTLs depleted of IFT20 by CRISPR/Cas9 gene editing and in the respective control CTLs edited with non-specific gRNAs (Fig. 1A). CTLs were generated from freshly purified CD8^+^ T cells activated with beads coated with anti-CD3 and anti-CD28 antibodies and used at days 5–7 post-activation, when they have fully differentiated to cytotoxic effectors [14]. Since LG biogenesis occurs after CD8^+^ T cell activation, IFT20 was knocked down prior to the onset of this process by nucleofection with the gRNAs and Cas9 at day 0 and IFT20 downregulation was tested at day 5 of differentiation (Fig. 1A).

The MPR recycling assay relies on a pool of MPRs that follow the default secretory pathway and are thus exposed at the plasma membrane, where they can capture and internalize M6P-tagged proteins from the extracellular milieu [10]. CTLs were incubated with an anti-MPR antibody at 37 °C for 4 h to allow for binding and internalization. Cells were then washed to remove unbound anti-MPR antibody, permeabilized, and MPR-antibody complexes were labelled with fluorescently tagged secondary antibodies (Supplementary Fig. 1A). CTLs were co-stained for the TGN marker TGN38 and visualized by confocal microscopy (Fig. 1B). Co-localization analyses showed a reduction in the co-localization of internalized MPRs with TGN38 in IFT20 KO CTLs compared to controls (Fig. 1B), indicating that retrograde trafficking of the MPR to the TGN is impaired in IFT20-deficient CTLs. These findings are consistent with our previous results, which revealed a defect in MPR retrograde trafficking in Jurkat and primary T cells, without affecting its expression on the plasma membrane [13].

To analyze GZMB intracellular localization in IFT20-deficient CTLs, we co-transfected IFT20-specific siRNAs and a GZMB-mCherry-encoding construct in day 2 CTLs (IFT20 KD) (Fig. 1C). Consistent with the role of the MPR pathway in the transport of the GZMs to LGs [12] and with the defect in retrograde MPR trafficking observed in IFT20 KO CTLs (Fig. 1B), we found an accumulation of GZMB in the TGN38^+^ compartment in IFT20 KD CTLs (Fig. 1D). Accordingly, the co-localization of GZMB with the lysosome/LG marker LAMP1 (Fig. 1E) as well as with the LG component PRF1 (Fig. 1F) was decreased in IFT20 KD CTLs. Nonetheless, the co-localization of PRF and LAMP1 was unaffected (Fig. 1G), further linking the defect in GZMB trafficking to IFT20 deficiency. In agreement with the MPR-dependent LG localization of GZMB [12], the co-localization analysis of GZMB with TGN38 and LAMP1 in MPR-deficient CTLs (MPR KD) recapitulated the alterations observed in IFT20 KD CTLs (Supplementary Fig. 2A–C).

**Fig. 2:**
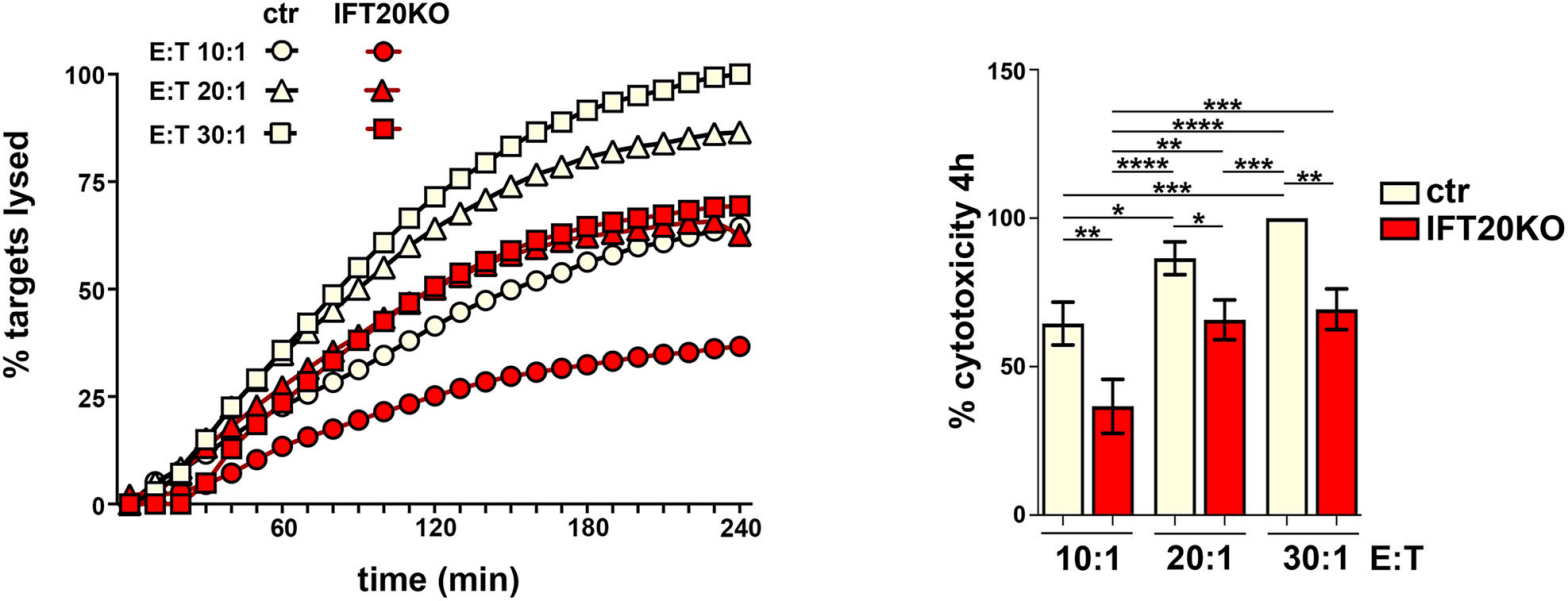
IFT20 is required for CTL-mediated killing. Real-time calcein release-based killing assay. Control or IFT20 KO CTLs were co-cultured with SAg-loaded Raji B cells at the target:CTL ratios indicated, and target cell killing was measured every 10 min for 4 h as reported in the kinetic graph (left). Quantification of the percentage of target cell death at the endpoint of the procedure (4 h) is shown on the right graph. The data refer to at least 3 independent experiments performed in duplicate and are reported as mean fold ± SD, with target cell lysis at the highest target:CTL ratio of the control sample set at 100% (ANOVA). *P < 0.05; **P < 0.01; ***P < 0.001; ****P < 0.0001.

These results indicate a defect in GZMB exit from the TGN and targeting to the LG compartment in IFT20-deficient CTLs, which may result in dysfunctional LGs and compromise their killing ability. To test this hypothesis, we carried out cytotoxicity assays, using as effector cells control or IFT20 KO CTLs and as targets Raji B cells pulsed with a mix of the staphylococcal superantigens SEA, SEE and SEB to allow for the polyclonal activation of T cells differing in their antigen specificity. Target cells were loaded with the cell-permeable probe calcein-AM, that becomes fluorescent through the activity of cellular esterases and is released following cell death [15]. IFT20 deficiency in CTLs led to an impairment in target cell killing at all effector:target cell ratios tested (Fig. 2), indicating that IFT20 is required for CTL-mediated cytotoxicity. Notably, also MPR KD CTLs showed impaired cytotoxic capability (Supplementary Fig. 2D).

### IFT20 deficiency is associated with LG abnormalities and abnormal transcription of the genes encoding LG components

**Fig. 3:**
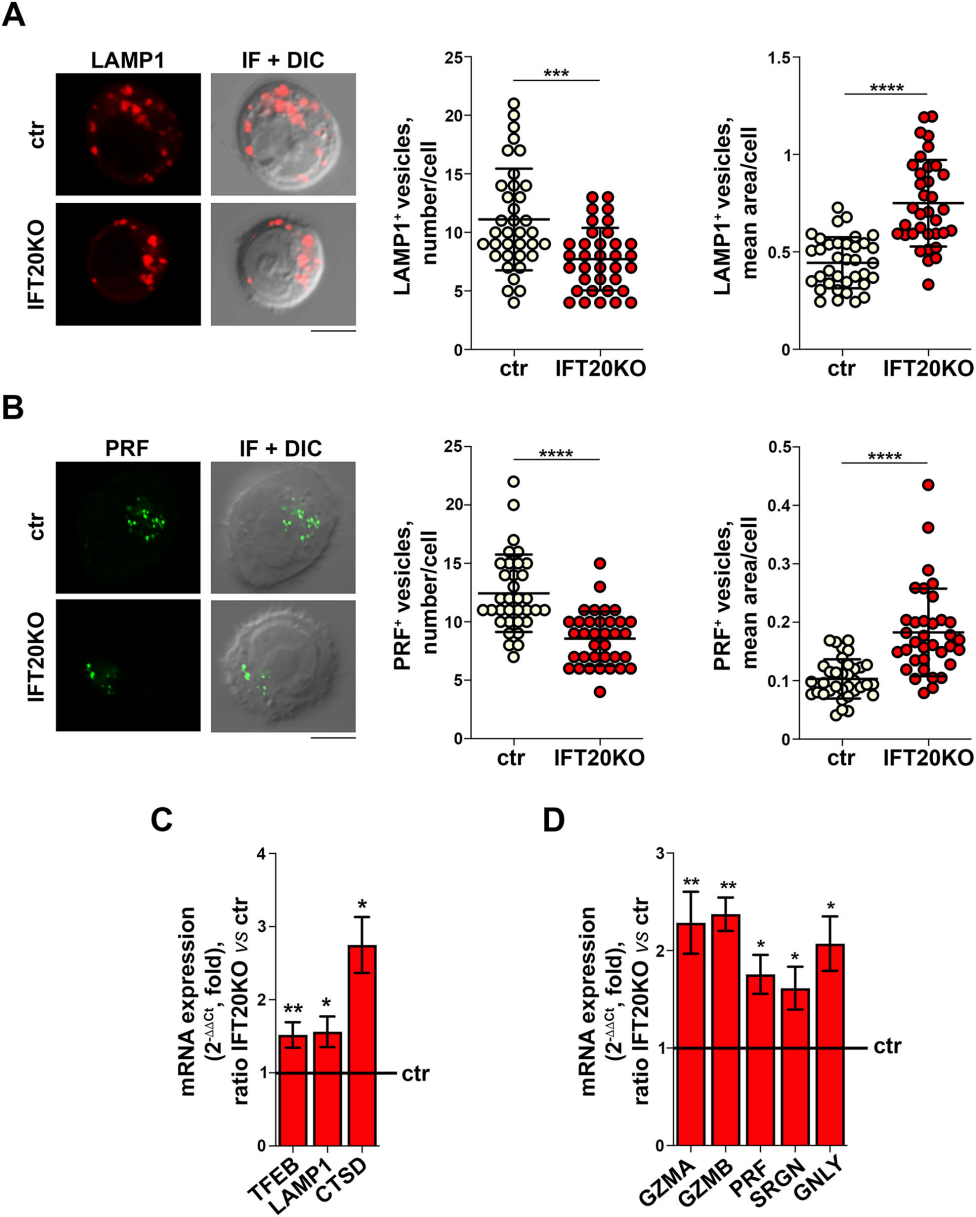
IFT20 depletion is associated to LG abnormalities in CTLs. Immunofluorescence analysis of LAMP1 (**A**) or PRF (**B**) in ctr and IFT20 KO CTLs. Representative images (medial optical sections) are shown. Scale bars: 5 μm. The graphs show the quantification of the number of LAMP1^+^ or PRF^+^ vesicles/cell (left) and their average size (μm^2^) (right) (≥35 cells from 3 independent experiments; mean ± SD, unpaired *t* test). RT-qPCR analysis of TFEB and TFEB-regulated (**C**) and LG component (**D**) genes in control and IFT20 KO CTLs (*n* ≥ 3; one sample *t* test). The relative abundance of gene transcripts was determined on duplicate samples using the ΔΔCt method and was normalized to human 18S. The data (mean ± SD) are expressed as normalized fold expression in IFT20 KO *versus* control, with the expression in control cells set for each gene as 1 (black line). *P < 0.05; **P < 0.01; ***P < 0.001; ****P < 0.0001.

Defective MPR trafficking and the resulting failure of the acid hydrolases to reach the lysosomes have been associated with abnormalities in the number and morphology of lysosomes, which decrease in number while increasing in size as they become engorged with undigested material [16]. We reasoned that LGs might harbor similar abnormalities in CTLs since, in addition to their cytotoxic function, LGs also subserve the function of lysosomes in these cells [17]. Consistent with this hypothesis, analysis of LGs in control and IFT20 KO CTLs stained for LAMP1 or PRF showed that IFT20 deficiency was associated with a reduction in the number and a concomitant increase in the size of LGs (Fig. 3A, B).

Lysosome dysfunction triggers a compensatory response whereby cells attempt to restore lysosome function by coordinately upregulating the transcription of the genes encoding the various lysosomal components [18, 19]. This transcriptional program, known as the CLEAR gene network, is regulated by the lysosome master transcription factor TFEB, which also regulates its own transcription [11, 18, 19]. In agreement with the lysosomal nature of LGs, RT-qPCR analysis showed that the expression of known members of the CLEAR network, namely LAMP1, CTSD and TFEB, was upregulated in IFT20 KO CTLs (Fig. 3C). Strikingly, an increase in the mRNA levels of key LG components, including the GZMs A and B, PRF, SRGN and the toxic molecule granulysin (GNLY), was also observed (Fig. 3D). Similar to IFT20 KO cells, MPR KD CTLs showed an upregulation in the transcription of the CLEAR gene network components (Supplementary Fig. 2E), as expected from the role of MPR in lysosome biogenesis [20]. Similar to IFT20 KO, MPR-deficient CTLs also upregulated the transcription of some LG components (Supplementary Fig. 2F). These results suggest the intriguing possibility that in CTLs the genes encoding the LG components are co-regulated with the ones belonging to the lysosomal CLEAR network.

### LG biogenesis is regulated by the transcription factor TFEB

**Fig. 4:**
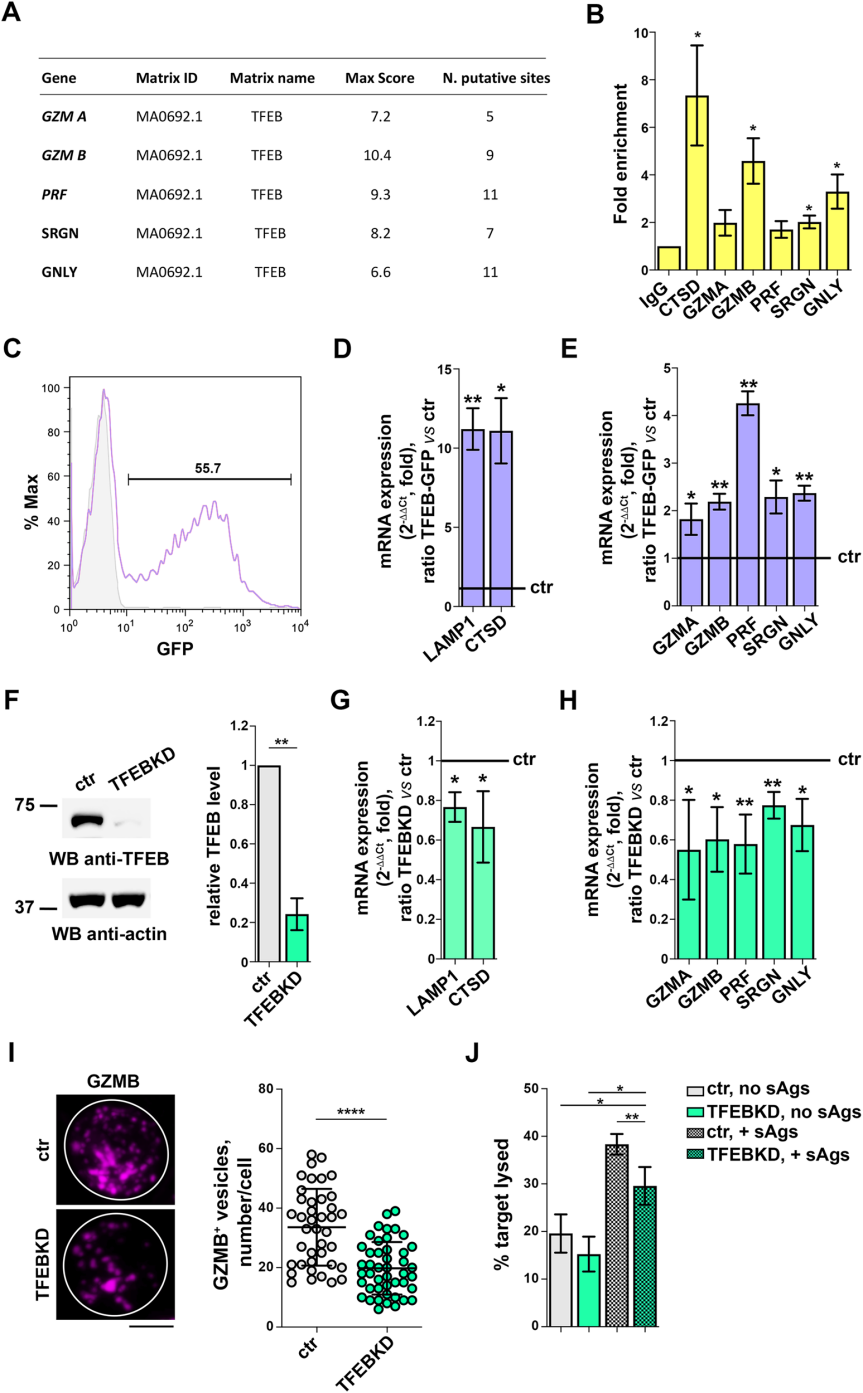
LG biogenesis is regulated by the transcription factor TFEB. **A** Predicted TFEB binding sites on the promoter regions of *GZMA*, *GZMB*, *PRF*, *SRGN* and *GNLY*. **B** ChIP analysis of TFEB binding to the promoter regions of *GZMA*, *GZMB*, *PRF*, *SRGN* and *GNLY*. **C** Representative flow cytometry profile of anti-GFP staining in TFEB-GFP expressing CTLs 24 h post transfection. The profile of unstained samples is shown in grey. **D**, **E** RT-qPCR analysis of TFEB-regulated (D) and LG component (E) genes in CTLs transiently transfected with a construct encoding GFP-tagged TFEB or with the respective GFP empty vector (*n* ≥ 3; one-sample *t* test). The relative abundance of gene transcripts was determined on duplicate samples using the ΔΔCt method and was normalized to human 18S. The data (mean ± SD) are expressed as normalized fold expression in TFEB-GFP *versus* control samples, with the expression in control cells set for each gene as 1 (black line). **F** Immunoblot analysis of TFEB in representative matched ctr and TFEB KD CTLs with respective loading control (actin). The migration of molecular mass markers is indicated. The quantification of TFEB in CTL lysates is reported in the graph (n = 3; mean fold ± SD, one-sample *t* test). RT-qPCR analysis of TFEB-regulated (**G**) and LG component (**H**) genes in control or TFEB KD CTLs (*n* ≥ 3; one-sample *t* test). The relative abundance of gene transcripts was determined on duplicate samples using the ΔΔCt method and was normalized to human 18S. The data (mean ± SD) are expressed as normalized fold expression in TFEB KD *versus* control samples, with the expression in control cells set for each gene as 1 (black line). **I** Immunofluorescence analysis of GZMB in ctr and TFEB KD CTLs. Representative images (medial optical sections) are shown. Scale bar: 5 μm. The graph shows the quantification of the number of GZMB^+^ vesicles/cell (≥35 cells from 3 independent experiments; mean ± SD, unpaired *t* test). **J** Flow cytometry analysis of cytotoxicity of CFSE-stained control or TFEB KD CTLs co-cultured with Raji cells loaded with SAg at the 1:10 target:CTL ratio for 4 h. The histogram shows the percentage (%) of target cells lysed (*n* = 3; ANOVA). *P < 0.05; **P < 0.01; ****P < 0.0001.

The co-regulation of the expression of genes encoding the lysosomal and LG components suggested the hypothesis that the latter may be also regulated by TFEB. To elucidate whether the LG-related genes are direct targets of TFEB we carried out an *in-silico* analysis using the JASPAR software, focusing on ~2 kb regions upstream of the *GZMA*, *GZMB*, *PRF*, *SRGN* and *GNLY* transcription start sites. This analysis revealed the presence of putative TFEB binding sites in all investigated promoters (Fig. 4A). To validate the prediction of the in silico analysis, we carried out a ChIP analysis on day 2 CTLs, using as a positive control the CLEAR gene *CTSD*. PCR amplification of the DNA pulled down by ChIP using an anti-TFEB antibody revealed the binding to the *GZMA*, *GZMB*, *PRF*, *SRGN* and *GNLY* promoters (Fig. 4B), supporting the notion that the LG and lysosome biogenesis programs are co-regulated by TFEB.

To test the potential role of TFEB in LG biogenesis we transfected CTLs with a construct encoding EGFP-tagged TFEB (Fig. 4C). As expected, TFEB overexpression led to a strong upregulation in the expression of CLEAR network genes, as assessed by quantifying LAMP1 and CTSD mRNA (Fig. 4D). Consistent with our hypothesis, the genes encoding GZMs A and B, PRF1, SRGN and GNLY were also upregulated in TFEB-overexpressing CTLs, albeit to a lesser extent compared to the lysosomal genes (Fig. 4E).

To further consolidate the role of TFEB in LG biogenesis we analyzed CTLs depleted of TFEB by RNA interference (Fig. 4F). TFEB deficiency led to a decrease in the expression of the CLEAR and LG-related genes (Fig. 4G, H), which was paralleled by a decrease in the number of LGs (Fig. 4I). Consistent with the reduced complement of LGs, killing was impaired in TFEB-deficient CTLs (Fig. 4J). Hence the genes encoding the cytotoxic components of the LGs belong to an extended CLEAR network, regulated by TFEB, operational in CTLs.

### IFT20 regulates TFEB function in CTLs by modulating the activity of mTOR

**Fig. 5:**
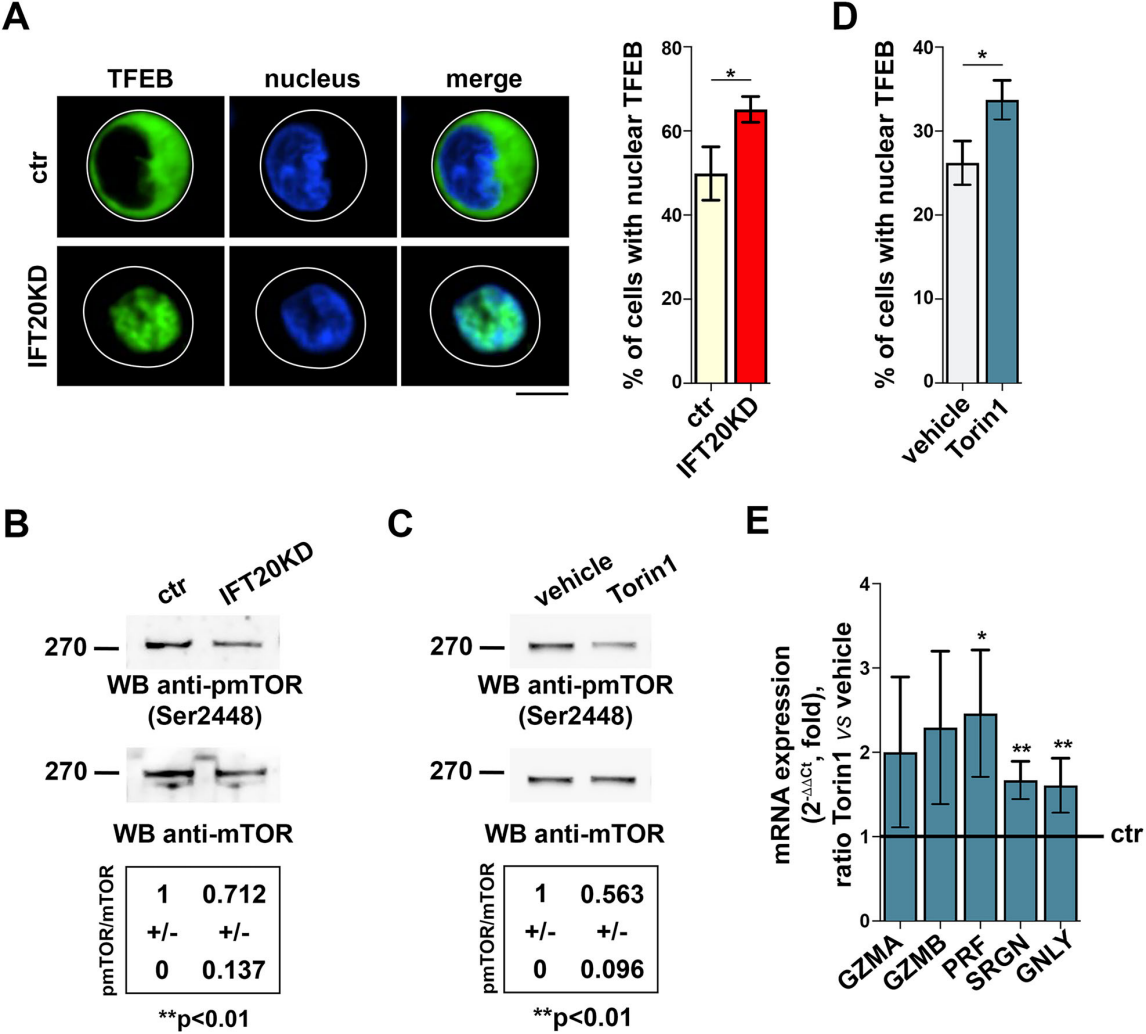
IFT20 regulates the mTOR-dependent activity of TFEB in CTLs. Immunofluorescence analysis of GFP-tagged TFEB in ctr and IFT20 KD CTLs (**A**) or Torin1-treated CTLs (**D**). The graphs show the quantification of nuclear TFEB in IFT20 KD (**A**) and Torin1-treated (3 h) (**D**) CTLs compared to control cells (*n* = 3; mean ± SD, unpaired *t* test). Representative images (medial optical sections) are shown. Scale bar: 5 μm. Immunoblot analysis of pmTOR in representative matched ctr and IFT20 KD (**B**) or Torin1-treated (**C**) CTLs. mTOR was used as loading control. The migration of molecular mass markers is indicated. The quantifications of pmTOR in CTL lysates are reported in the tables (*n* ≥ 3; mean fold ± SD, one-sample *t* test). **E** Quantitative RT-PCR analysis of LG component genes in CTLs treated with Torin1 for 24 h (*n* ≥ 4; one-sample *t* test). The relative abundance of gene transcripts was determined on duplicate samples using the ΔΔCt method and was normalized to human 18S. The data (mean ± SD) are expressed as normalized fold expression in treated *versus* control samples, with the expression in control cells set for each gene as 1 (black line). *P < 0.05; **P < 0.01.

TFEB-dependent gene transcription is dictated by its subcellular localization. In basal conditions, TFEB is retained in the cytosol, at the lysosome surface, through phosphorylation by mTORC1 [21, 22]. Lysosome dysfunction or cell starvation leads to the inactivation of mTORC1 and the concomitant activation of phosphatases such as calcineurin and PP2A, which dephosphorylate TFEB, allowing for its nuclear translocation [23–25]. Consistent with the lysosome defects resulting from impaired MPR recycling in IFT20 KO CTLs, nuclear translocation of TFEB was enhanced in IFT20-deficient CTLs, as assessed by imaging TFEB in CTLs depleted of IFT20 by RNA interference and transfected with a construct encoding GFP-tagged TFEB (Fig. 5A). Additionally, immunoblot analysis showed a reduction in the active form of mTOR in IFT20 KD CTLs compared to CTLs transfected with control siRNAs (Fig. 5B).

To establish whether the defect in mTOR activity observed in IFT20-deficient CTLs may be causal to the enhanced nuclear translocation of TFEB, we imaged TFEB in CTLs transfected with the TFEB-GFP encoding construct and treated CTLs with the mTOR inhibitor Torin1 [13]. Treatment with the inhibitor phenocopied the TFEB abnormalities observed in IFT20 KD cells (Fig. 5C, D). Consistent with the enhanced nuclear translocation of TFEB, a RT-qPCR analysis showed that the expression of the genes encoding *GZMA*, *GZMB*, *PRF*, *SRGN* and *GNLY* was upregulated in Torin1-treated CTLs (Fig. 5E). Collectively, these data suggest that the lysosome dysfunction caused by defective MPR trafficking in IFT20-deficient CTLs may lead to mTOR inactivation and thus TFEB dephosphorylation, thereby facilitating its nuclear translocation and the transcription of its target genes.

## Discussion

The trafficking pathways and molecular determinants that regulate the targeting of the lytic effectors of CTLs to LGs and their retention therein are as not fully described yet, particularly in the light of the emerging diversity among LGs [26]. Here we identify the ciliary protein IFT20, that we had previously implicated in lysosome biogenesis [13], as a new regulator of LG biogenesis, to which it contributes by orchestrating the MPR-mediated transport of a key LG component, GZMB, to the lysosomal compartment. Additionally, we highlight a link between IFT20 and TFEB, the master transcription factor that coordinates the expression of the lysosome biogenesis gene network [18, 19], which we show to promote the expression of LG-critical genes, highlighting the existence of an extended CLEAR network regulated by TFEB that coordinates the biogenesis of these LROs in CTLs.

We previously reported that the ciliary protein IFT20 regulates IS assembly in T cells alongside other ciliogenesis-related proteins [27–29], revealing an unexpected similarity between these signaling and secretory platforms [30–33]. IFT20 coordinates vesicular trafficking to sustain TCR signaling [28] by guiding its transit from early to recycling endosomes [27]. As the sorting hub for endocytic proteins [34], early endosomes coordinate not only recycling of endocytosed proteins to the cell surface, but also recycling of proteins participating in the machinery that regulates retrograde trafficking to intracellular vesicular compartments, of which a key one is the MPR. The MPR, after delivering M6P-tagged lysosomal hydrolases from the TGN to early endosomes, relies on retromer-mediated dynein-dependent transport for its retrograde trafficking back to the TGN [10, 16]. Positioned at early endosomes, IFT20 promotes both TCR and MPR recycling by coupling the MPR to dynein in both Jurkat and primary human T cells [13]. Here we show that IFT20 is required for MPR recycling to the TGN also in primary human CD8^+^ T cells differentiated to CTL. As a result, the MPR-dependent targeting of GZMB to LGs is impaired in IFT20-deficient CTLs, leading to the biogenesis of dysfunctional LGs that are unable to efficiently kill target cells. These data not only identify IFT20 as a new player in the vesicular trafficking pathways orchestrating LG biogenesis, but also provide evidence that the role of IFT20 in lysosome biogenesis through the regulation of MPR recycling is conserved in CTLs to generate the LGs, consistent with the lysosomal origin of these organelles.

LGs were initially identified as secretory lysosomes featuring a dense core enriched in cytotoxic molecules, the GZMs and PRF, packed onto the proteoglycan SRGN. Surrounding the dense core, LGs harbor vesicles that are decorated by membrane proteins, including the TCR and FasL [35–37], highlighting the multivesicular body (MVB) origin of these LROs [1]. The presence on the membrane of these vesicles of the MPR, which is lost at the final steps of lysosome maturation, and of lysosomal hydrolases in the surrounding matrix [17], suggests that LGs derive from a post-MVB, pre-lysosomal compartment. Other LG morphologies, spanning from MVB-like organelles to LGs consisting almost exclusively of a dense core, had been interpreted as maturation intermediates [7]. While this notion may still stand, this unified picture of LGs has substantially evolved over the years with the expanding heterogeneity of these organelles. Proteomic analyses of LGs purified from human CTLs as well as NK cells provided evidence of the co-existence of two distinct populations of differently sized LGs, of which the larger enriched in FasL and the smaller in GZMs and PRF1 [38], that have been demonstrated to have different requirements for release [39, 40]. Additionally, a recent study on mature LGs immunopurified from mouse CTLs has demonstrated the presence of two types of GZMB^+^PRF^+^ granules, the single core granules (SCG) and the multicore granules (MCG), differing in size, morphology of the dense core and protein composition, with the SCGs enriched in lysosomal proteins and the MCGs in trafficking regulators [26]. Interestingly, MCGs are selectively enriched in TSP-1, a component of the Supramolecular Attack Particles (SMAP), a new class of non-membranous nanoparticles with killing activity, consisting of a core of GZMs and PRF encased in a glycoprotein shell, that are released by activated CTLs at the IS [41]. The selective association of TSP-1 with MCGs highlights this population of LGs as the compartment where SMAPs are assembled [26]. These findings suggest a diversity in the vesicular trafficking pathways exploited not only for the biogenesis of the individual FasL^+^ and GZMB^+^ LGs that trigger the two main apoptosis pathways in target cells, but also for the biogenesis of the two distinct subpopulations of GZMB^+^ LGs. While our data suggest that IFT20 regulates the MPR-dependent transport of GZMB to LGs prior to their bifurcation to SCGs and MCGs, how these pathways are built, how they interrelate, and where they diversify, are open questions. A critical re-evaluation of the cytotoxicity defects in primary immunodeficiencies, together with the pathways that coordinate the biogenesis of LROs in other cell types, may provide important clues.

Lysosome biogenesis is coordinated by the master transcription factor TFEB, which regulates the expression of the genes encoding all functional components of the lysosomes as well as its own transcription [19]. Here we show that TFEB modulation affects LG biogenesis in CTLs, with an upregulation of the genes encoding LG components in TFEB-overexpressing CTLs and a downregulation in TFEB-depleted CTLs, implicating TFEB in the transcriptional program that orchestrates LG biogenesis. Consistent with this notion, we have identified functional TFEB binding sites in the promoter of genes encoding critical LG components, namely *GZMA*, *GZMB*, *PRF*, *SRGN* and *GNLY*. In agreement with a role for TFEB in LG biogenesis, TFEB deficiency leads to abnormalities in CTL-mediated killing. Hence the LG-relevant genes emerge as part of an extended TFEB-regulated CLEAR network in CTLs. Furthermore, we have recently implicated TFEB in the expression of the critical pioneer transcription factors RUNX3 and T-BET, which are essential for orchestrating the coordinated transcription of genes encoding the cytotoxic machinery [42]. Together with the data presented in the present report, these findings identify TFEB as a novel transcriptional regulator of the CTL differentiation program.

Our finding that TFEB and the CLEAR network are upregulated in IFT20-deficient cells highlights a functional link between IFT20 and TFEB. TFEB is finely tuned by the activity of the kinase mTOR, which phosphorylates TFEB preventing its nuclear translocation [21, 22]. Under conditions that alter lysosome function, which can be either physiological or pathological, such as defective transport of the lysosomal hydrolases, TFEB is dephosphorylated and migrates to the nucleus to activate de novo lysosome biogenesis [43]. The upregulation in the TFEB-dependent expression of lysosomal and LG components in IFT20-deficient CTLs is paralleled by a decrease in the basal activity of mTOR, which may be related to the defective tonic TCR signaling previously reported in IFT20-deficient T cells [13]. Additionally, inhibition of mTOR recapitulates the abnormalities in subcellular TFEB localization and in the expression of the LG components observed in IFT20-deficient cells, supporting a causal, lysosome-related link between IFT20 and TFEB in LG biogenesis.

In conclusion, our data demonstrate that, by promoting MPR retrograde transport, IFT20 regulates the biogenesis of the GZM^+^ LGs, and provide evidence of a transcriptional program coordinated by TFEB that orchestrates the dual lysosomal and cytotoxic function of LGs. The data suggest a potential implication of IFT20 and TFEB defects in diseases characterized by impaired CTL function, of which prominent ones are primary immunodeficiencies and cancer [44–46]. They also suggest the intriguing possibility that IFT20 and TFEB might be implicated in the biogenesis of other LROs, such as pigment cell melanosomes, endothelial cell Weibel-Palade bodies or platelet alpha granules [47].

## Materials and methods

### Cells, stimulations, and antibodies

Peripheral blood CD8^+^ T cells were isolated from anonymous healthy donors obtained from the Siena University Hospital blood bank. The study was approved by the local ethics committee (Siena University Hospital). Informed consent was obtained from blood donors by the physician in charge of the Siena University Hospital blood bank. Samples were anonymized before distribution. Primary CD8^+^ T cells were isolated by negative selection through the RosetteSepTM Human CD8^+^ T Cell Enrichment Cocktail (StemCell technologies, Vancouver, Canada) and centrifugation over a buoyant density medium (Lympholyte Cell Separation Medium, Euroclone, Milan, Italy). Immediately after isolation, CD8^+^ T cells were resuspended in RPMI 1640 with 25 mM Hepes (Sigma-Aldrich, St. Louis, Missouri, USA), supplemented with 10% BCS (Bovine Calf Serum, Hyclone, Logan, Utah, USA) inactivated at 56 °C for 30 min, 20 U/mL Penicillin (Sigma-Adrich) and 1X non-essential amino acids (MEM non-essential amino acids solution 100X, Gibco, Waltham, Massachusetts, USA). Freshly isolated CD8^+^ T cells were differentiated in vitro to CTLs by incubation for 48 h with anti-CD3/CD28 mAb-coated magnetic beads (Dynabeads Human T-activator CD3/CD28, Gibco) and 50 U/mL IL2 (human Interleukin-2, Miltenyi Biotech, Bergisch Gladbach, Germany). Mature CTLs were employed in the assays from day 5–7 of differentiation [14]. Other cells used were Raji B cells (CCL-86, ATCC, Manassas, Virginia, USA), cultured in RPMI 1640 supplemented with 7.5% BCS and 20 U/ml Penicillin. The cells were routinely tested for mycoplasma contamination and confirmed to be mycoplasma free. Cells were incubated at 37 °C and 5% CO_2_.

To inhibit mTOR activity, CTLs were treated for the indicated times with 250 nM Torin1 (#4247, Tocris, Bristol, UK).

All primary commercial antibodies used in the assays are listed in Table S1, together with information about the dilutions used for flow cytometry, immunoblotting and immunofluorescence. Polyclonal anti-IFT20 antibodies [48] were kindly provided by G. Pazour. Secondary horseradish peroxidase-labelled antibodies were purchased from Jackson ImmunoResearch Laboratories (West Grove, Pennsylvania, USA) and Alexa Fluor 488-, 555- and 647-labeled secondary antibodies from ThermoFisher Scientific (Waltham, Massachusetts, USA).

### CRISP-R/Cas9-mediated gene editing, siRNA-mediated gene silencing, generation of T cell transfectants

Specific single guide RNAs (gRNAs) directing the nuclease Cas9 to either IFT20 or GFP as negative control (Table S2) were designed using the web-based tool CRISPOR [49]. A gRNA transcription template was prepared by PCR amplification using the PX458 construct as a template and the primers listed in Table S2, and then transcribed in vitro using the HiScribe T7 high yield RNA synthesis kit (NEB, Ipswich, Massachusetts, USA). gRNAs were purified by RNA clean & concentration (Zymo Research, Irvine, California, USA). Freshly isolated CD8^+^ T cells were transfected using the Human T cell nucleofector kit and the program V-024 of the Nucleofector II system (Amaxa Biosystems, Euroclone, Milan, Italy) for unstimulated T cells, with ribonucleoprotein complexes formed mixing 10 μg of Alt-R Cas9 Nuclease V3 protein (IDT, Coralville, Iowa, USA) and 6 μg of gRNA. Cells were then activated with anti-CD3/CD28 magnetic beads in AIM V culture medium (ThermoFisher Scientific) supplemented with 10% BCS and 500 U/ml of human IL-2 for 48 h. After removal of the beads, cells were expanded in AIM V 10% BCS with 50 U/ml human IL-2. Transfected blasts were tested for gene editing by immunoblotting 5 days after isolation and used for the assays from day 5 to 7.

For RNAi-mediated IFT20 silencing, CTLs were transiently transfected at day 6 of differentiation using the Human T cell nucleofector kit and the program T-023 of the Nucleofector II system (Amaxa Biosystems) for activated cells with human IFT20-specific endoribonuclease-prepared siRNAs (esiRNA)(Sigma-Aldrich) and unrelated control *Renilla* luciferase (RLUC) esiRNA (Sigma-Aldrich). Alternatively, CTLs at day 2 of differentiation were co-transfected with IFT20-specific siRNAs and a GZMB-mCherry-encoding construct, kindly provided by Prof. Jens Rettig [50]. Cells were used 24 h post-transfection. All samples were tested by immunoblotting to check the efficiency of IFT20 knockdown.

To modulate TFEB expression or function CTLs at day 5 of differentiation were transiently transfected using the Human T cell nucleofector kit and the program T-023 of the Nucleofector II system (Amaxa Bioystems) for activated T cells, with 1 μg × 10^6^ cells of pEGFP-N1 or pEGFP-N1-TFEB plasmids. Transfected cells were gently resuspended in the culture medium with 500 U/ml IL2 and analyzed 36 h post transfection. The pEGFP-N1-TFEB plasmid was kindly provided by D. Medina. Alternatively, for RNAi-mediated TFEB silencing CTLs were transiently transfected at day 2 of differentiation using the Human T cell nucleofector kit and the program T-023 of the Nucleofector II system (Amaxa Biosystems) for activated cells with human TFEB-specific (#FE5L009798000010) or negative control (#FE5D0018101020) siRNAs (Dharmacon, Lafayette, Colorado, USA) (150 ng × 10^6^ cells). Cells were used 24 h post transfection. All samples were tested by immunoblotting to check the efficiency of TFEB knockdown.

### Cell lysis and immunoblotting

Cells (2 × 10^6^/sample) were lysed in 1%(v/v) Triton-X100 in 20 mM Tris–HCl (pH 8), 150 mM NaCl in the presence of Protease inhibitor Cocktail Set III (Calbiochem, Merck, Darmstadt, Germany) and the phosphatase inhibitor sodium orthovanadate (Sigma-Aldrich) for 5 min on ice. Protein extracts from post-nuclear supernatants were quantified using the Quantum protein assay kit (Euroclone) and denatured in 4x Bolt SDS sample buffer (Invitrogen, Waltham, Massachusetts, USA) supplemented with 10x Bolt sample reducing buffer (Invitrogen) for 5 min at 100 °C. Proteins (10 μg) were subjected to SDS-Page on Bolt Bis-Tris mini protein gels (Invitrogen) and transferred to nitrocellulose (GE HealthCare, Chicago, Illinois, USA) under wet conditions. Blocking was performed in 5% non-fat dry milk in PBS containing 0.2% Tween 20 (Sigma-Adrich). Membranes were incubated in primary antibodies for 1–3 h at room temperature (20–25 °C) or overnight at 4 °C, followed by incubation in 20 ng/ml HRP-conjugated secondary antibodies (Jackson ImmunoResearch Laboratories) for 45 min at room temperature. Secondary antibodies were detected using SuperSignal west pico plus chemiluminescent substrate (Life Technologies, Waltham, Massachusetts, USA). For quantification, immunoblot membranes were scanned using a laser densitometer (DuoScan T2500, Agfa, Mortsel, Belgium) or Alliance Q9-Atom chemiluminescence imaging system (Uvitec, Cambridge, UK), and densitometric levels were measured using ImageJ software (National Institutes of Health, USA).

All antibodies were initially tested on full-length filters to check that the electrophoretic mobility of the proteins against which they were raised corresponded to the expected molecular mass and that they gave a clear signal above background. After that we cut the filters in order to have, on the same samples and on the same run, blots with different antibodies recognizing proteins with different molecular mass. This allowed for a more rigorous comparison of the relative intensity of each signal within samples. Full scans of the immunoblots shown in the figures are presented in Supplementary Material 2.

### Immunofluorescence and image analysis

Samples were seeded onto poly-L-lysine (Merck)-coated slides (ThermoFisher Scientific) and fixed in either methanol at -20 °C for 10 min or with 4% paraformaldehyde/PBS at room temperature for 15 min. After washing with PBS, samples were stained with primary antibodies at 4 °C overnight and then incubated at room temperature for 45 min with Alexa fluor 488-, 555- or 647-labeled secondary antibodies and mounted with 90% glycerol/PBS. Nuclei were counterstained with Hoechst 33342 dye (ThermoFisher Scientific, #62249).

Confocal microscopy was carried out on a Zeiss LSM700 microscope (Carl Zeiss, Jena, Germany) using a 63x/1.40 oil immersion objective or a spinning disk confocal and super-resolution microscope (CSU-W1-SoRA Nikon, Tokyo, Japan), with 60x/1.49 oil objective. Detectors were set to detect the optimal signal below the saturation limits.

Co-localization analyses were performed on a medial optical section of single cells using ImageJ and the JACoP plugin to calculate Manders’ coefficient M1, which indicates the proportion of the green signal coincident with a signal in the red channel over its total intensity, and M2, which is defined conversely for red [51]. Manders’ coefficients range from 0 to 1, corresponding to non-overlapping images and 100% co-localization between both images, respectively.

The number and area of LAMP1^+^ and GZMB^+^ dots in IFT20 KO CTLs were determined by immunofluorescence. The area of vesicles was measured in medial optical sections by manual gating using the ImageJ software.

The number of GZMB^+^ dots in TFEB KD CTLs was determined by immunofluorescence analysis of 3D reconstruction of z-stack carried out on fixed samples at 200 nm steps using a Nikon CSU-W1-SoRA system (Nikon) with a 60X/1.49 NA oil immersion objective and a Photometrics BSI (Nikon). Bright spots were identified and counted on 3D images using the Nis-Elements AR 5.42.05 software (Nikon).

### MPR recycling assay

For MPR recycling assays, CTLs were incubated for 4 h at 37 °C to allow the recycling of antibody-tagged MPRs, which were detected after fluorochrome-labelled secondary antibody staining [13]. Briefly, cells were fixed in methanol at -20 °C for 10 min and, after washing with PBS, stained with primary antibodies anti-TGN38 at 4 °C overnight and then incubated at room temperature for 45 min with Alexa fluor 488- and 555-labeled secondary antibodies.

### Cytotoxicity assays

For real-time calcein release-based killing assay, Raji B cells were loaded with 500 nM calcein-AM (Invitrogen) in AIM V medium (ThermoFisher Scientific) with 10 mM Hepes at room temperature for 15 min, washed and plated into 96-well black plates with clear bottoms (BD Falcon, Franklin Lakes, USA). IFT20 KO and control CTLs were added at different ratios to 0.5 × 10^4^ settled target cells per well to measure killing at 37 °C. Triton X-100 (1%) was added to target cells alone to calculate maximal target cell lysis as control. Target cell lysis was measured every 10 min for 4 h. The decreased calcein fluorescence in target cells due to cell lysis was measured at 485 nm excitation wavelength and 528 nm emission wavelength in the bottom reading mode using a Synergy HTX multi-mode plate reader (BioTek, Milan, Italy). The fluorescence for the experimental condition was adjusted by the parameter g according to the live target cell control fluorescence. The g value was measured at time zero: *g* = *F*_live_(0)/*F*_exp_(0). Cytotoxicity was calculated based on the loss of calcein fluorescence in target cells using the equation: % target cell lysis = (*F*_live_ − g × *F*_exp_)/(*F*_live_ − *F*_lyse_) × 100, where F_live_ is the fluorescence of target cells alone, F_exp_ are CTL-APC samples and F_lyse_ is the maximal target cell lysis. All experiments were performed in duplicate and averaged to obtain one dataset for each donor. The maximal CTL-induced target cell killing was assigned to the higher CTL-APC ratio, on which the values for the other samples were normalized [52].

For flow cytometry analysis of cytotoxicity, T cells were stained with 0.5 μM CFSE for 8 min at RT and co-cultured with Raji cells loaded with SAgs at the indicated target:CTL ratios for 4 h. Cells were stained with propidium iodide (PI) prior to processing for flow cytometry. Analyses were carried out gating on CFSE^-^/PI^+^ cells.

### Chromatin immunoprecipitation

ChIP assays for the analysis of TFEB binding to the promoters of *GZMB, GZMA, PRF1, SRGN* and *GNLY* was carried out using MAGnify Chromatin Immunoprecipitation System (ThermoFisher Scientific). 2 × 10^6^ CTLs at day 5 of differentiation were crosslinked with 1% formaldehyde for 10 min at room temperature. Cells were lysed and sonicated 10 times for 10 s to obtain average DNA fragment sizes of 300–500 bases. Immunoprecipitations were carried out using 2 μg of either anti-TFEB or control rabbit IgG. The immunoprecipitated DNA fragments were quantitated by RT-qPCR as described below. The primer sets used for the analyses are listed in Table S2.

### RNA purification and RT-qPCR

RNA was purified from CTLs using the RNeasy plus mini kit (Qiagen, Hilden, Germany), reverse transcribed to single-strand cDNA using the iScript cDNA synthesis kit (Bio-Rad, Hercules, California, USA) and analyzed by Real-time quantitative PCR (RT-qPCR) on 96-well optical PCR plates (Sarstedt, Nümbrecht, Germany) using the SsoFast EvaGreen supermix (Bio-Rad) and specific primers for human transcripts (listed in Table S2). The quantity of the transcripts of interest was determined using the ΔΔCt method and normalized to the housekeeping gene 18S.

### Flow cytometry

The transfection efficiency of the pEGFP constructs was evaluated by flow cytometric analysis of transfected CTLs fixed and permeabilized using Cyto-Fast Fix/Perm buffer set (Bio Legend, San Diego, California, USA), incubated on ice for 1 h with anti-GFP antibodies and then for 45 min with Alexa fluor 488-labeled secondary antibodies. Samples were acquired with a Guava easyCyte cytometer (Millipore, Burlington, Massachusetts, USA) and plotted using FlowJo software (TreeStar Inc., Ashland, Oregon, USA).

### Statistics and reproducibility

Sample size, based on commonly accepted practice in the field and constrained by available resources, and replicate number for each experimental group/condition are indicated in the figure legends. Statistical analyses were performed using Prism software (GraphPad Software, La Jolla, California, USA). Pairwise or multiple comparisons of values with normal distribution were carried out using Student’s *t* test (unpaired), one-sample *t* test (theoretical mean = 1) and one-way ANOVA, whereas values without Gaussian distribution were analyzed with Mann–Whitney test or Kruskal–Wallis test. The details of statistical analysis are indicated in the figure legends. Statistical significance was defined as: ****P ≤ 0.0001; ***P ≤ 0.001; **P ≤ 0.01; *P ≤ 0.05.

## Supplementary information

Supplementary tables, supplementary figures and legends

Original data_WB

Original data_qPCR

Reproducibility checklist

## Data Availability

Data generated in this study are available upon request from the corresponding author FF.
