# Supplementary material for: IFT20 regulates TFEB-dependent lytic granule biogenesis in cytotoxic T lymphocytes by orchestrating the MPR-dependent transport of granzyme B
Source: Cell Death Dis. 2025 May 19;16(1):398. doi: 10.1038/s41419-025-07727-5 (PMC12089405; doi:10.1038/s41419-025-07727-5)
Supplement: Supplementary file 1 — Supplementary tables, supplementary figures and legends [file 41419_2025_7727_MOESM1_ESM.pdf]

Supplementary figure legends

Figure S1. Schematic representation of the MPR recycling assay. Cells are incubated with an anti-MPR antibody (panel I) at 37°C for 4 h to allow internalization and recycling of antibody-tagged MPRs (yellow), which are then detected by confocal microscopy after staining with fluorochrome-labelled secondary antibodies (magenta) (panel II).

Figure S2. The MPR is required for LG biogenesis and CTL activity. (A) Immunoblot analysis of MPR in representative matched ctr and MPR KD CTLs with respective loading control (actin). The migration of molecular mass markers is indicated. The quantification of MPR in CTL lysates is reported in the table (n=4; mean fold ± SD, one sample t test). **(B,C)** Immunofluorescence analysis of GZMB-mCherry and TGN38 (B) or LAMP1 (C) in MPR KD CTLs. The graphs show the quantification using Mander's coefficient of the weighted colocalization of GZMB-mCherry and TGN38 (B) or LAMP1 (C) in either ctr or MPR KD CTLs (≥35 cells from 3 independent experiments; mean fold ± SD, unpaired t test). **(D)** Flow cytometry analysis of cytotoxicity of CFSE-stained ctr or MPR KD CTLs co-cultured with Raji cells loaded with SAg at the 1:10 target:CTL ratio for 4 h. The histograms show the percentage (%) of target cells lysed (n=7; ANOVA). **(E,F)** RT-qPCR analysis of TFEB and TFEB-regulated (E) and LG component (F) genes in ctr and MPR KD CTLs (n≥3; one sample t test). The relative abundance of gene transcripts was determined on duplicate samples using the $\Delta\Delta$Ct method and was normalized to human 18S. The data (mean ± SD) are expressed as normalized fold expression in MPR KD versus control, with the expression in control cells set for each gene as 1 (black line). *P < 0.05; **P < 0.01; ***P < 0.001; ****P < 0.0001.

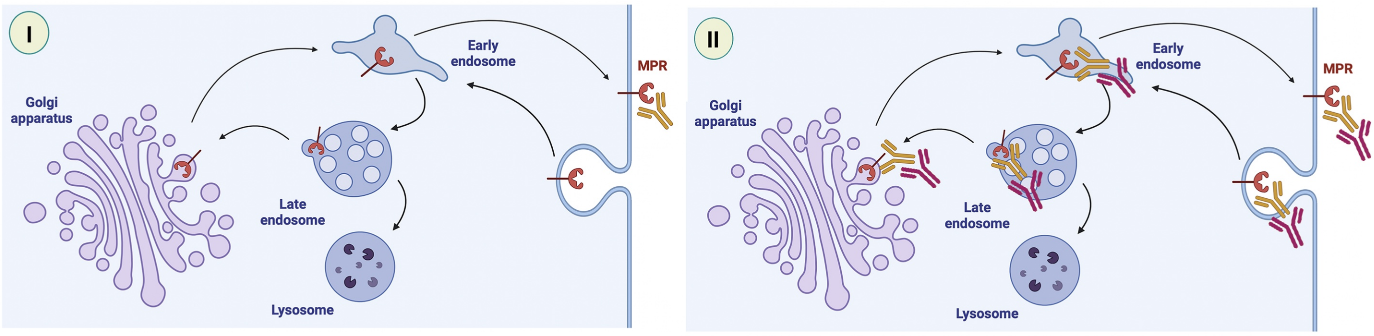

Supplementary Figure 1

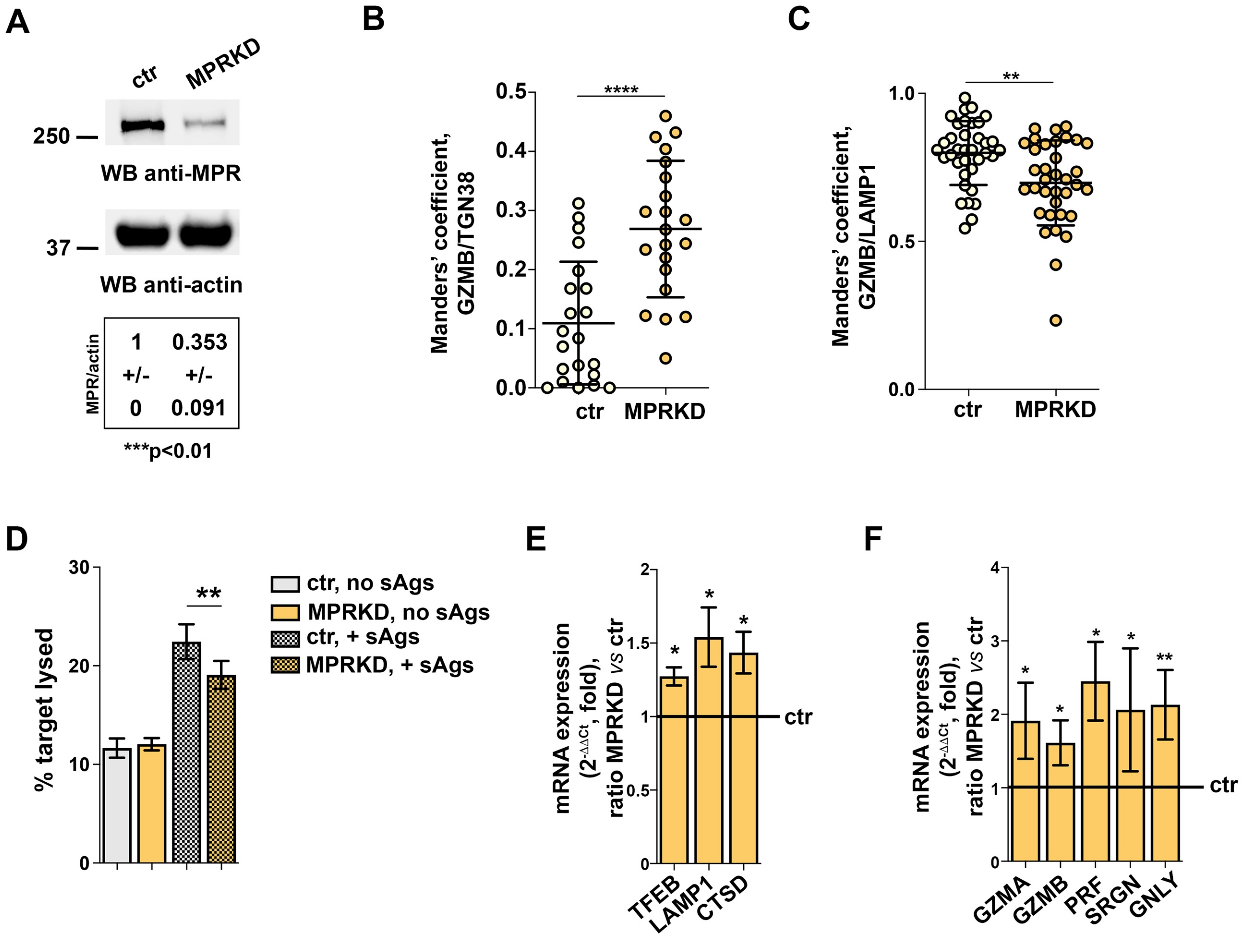

Supplementary Figure 2

Supplementary tables

Table S1. List of the primary antibodies used in this work

Antibody (anti-)	Host species	Company	Catalog number	Dilution WB	Dilution IF	Dilution FC
Actin	mouse	Millipore	#MAB1501	1:10,000	-	-
GFP	mouse	Invitrogen	#A11120	-	-	1:100
GZMB-FITC	mouse	BD Pharmingen	#560211	-	1:20	-
IFT20	rabbit	G.J. Pazour*	-	1:500	-	-
LAMP1	mouse	Millipore	#328602	-	1:400	-
LAMP1-647	mouse	BioLegend	#328612	-	1:150	-
MPR	rabbit	Abcam	#ab32815	-	1:400	-
mTOR	rabbit	Cell Signaling	#2972	1:1,000	-	-
pmTOR (Ser2448)	rabbit	Cell Signaling	#2971	1:1,000	-	-
PRF-488	mouse	BioLegend	#308108	-	1:50	-
RFP	rabbit	Rockland Immunochemicals	#600-401-379	-	1:100	-
TFEB	rabbit	Cell Signaling	#37785	1:1,000	-	-
TGN38	mouse	Santa Cruz Biotechnology	#sc-166224	-	1:300	-

*Program in Molecular Medicine, University of Massachusetts Medical School, Worchester, MA 01605, USA.

Table S2. List of the primers used in this work

Oligo name	Forward 5’-3’	Reverse 3’-5’	Description
Common reverse primer		AGCACCGACTCGGTGCCACT	gRNA production
GFP gRNA	ttaatacgactcactataggGGGCGAGGAGC<br>TGTTCACCGttttagagctagaaatagc		gRNA production
IFT20 gRNA	ttaatacgactcactataggGAGTGTAGCC<br>CTGCTTCACCCGttttagagctagaaatagc		gRNA production
IFT20 gRNA	GAGTGTAGCCCTGCTTCACCCG		gRNA sequence cloned into pSpCas9(BB)-2A-GFP plasmid
GZMA	AACCAGGAACCATGTGCCAA	GGCTTCCAGAATCTCCATTGC	qPCR primer
GZMA	AAGAAATGCAGGGGTCTCAGC	GAGCTGAGGATGTGGTCTCC	ChIP primer
GZMB	TCAAAGAACAGGAGCCGAC	TTGGCCTTTCTCTCCAGCTG	qPCR primer
GZMB	TGTCCAGAGAGCCACACTTC	TTTAACAGAATTGGGCACGGG	ChIP primer
PRF	CCTGCAGTCACAGCTACACA	GGGGCTCCAGTTAAGGCAA	qPCR primer
PRF	AAATCACACGGCTTCTGGGG	ATGAGCCCCAAAGTGTGACC	ChIP primer
SRGN	GACGAGAATCCAGGACTTGAA	GGGCAGATTCCTGTCAAGAG	qPCR primer
SRGN	GGGTTTCACGGTGTTAGCCA	TCTGGAGGACTAAGGCTCCA	ChIP primer

GNLY	GGATAAGCCCACCCAGAGAAG	ACAGATCTGCTGGGCAGTTT	qPCR primer
GNLY	AGACCACCCCTTCCTCCTTC	ACTCCAGCTCAAAACAAACAAACA	ChIP primer
TFEB	GGAGTACCTGTCCGAGACCT	GGGCTATTGGGAGCACTGTT	qPCR primer
LAMP1	CCGCGGTGTCTTCTTCGTG	TAGAGACAGCGGGCGTTACC	qPCR primer
CTSD	CCAGTGCTTCACAGTCGTCT	CACGTAGGTGCTGGACTTGT	qPCR primer
CTSD	GCAGCTTCCAGGTCATAGGG	CTGACCTCAGGTGATCTGCC	ChIP primer
18S	CGCCGCTAGAGGTGAAATT	CTTGGCAAATGCTTTCGC	qPCR primer
