# Supplementary material for: IFT20 regulates TFEB-dependent lytic granule biogenesis in cytotoxic T lymphocytes by orchestrating the MPR-dependent transport of granzyme B
Source: Cell Death Dis. 2025 May 19;16(1):398. doi: 10.1038/s41419-025-07727-5 (PMC12089405; doi:10.1038/s41419-025-07727-5)

Original Images for Western Blots

The main figure to which they refer is indicated in the upper-right area of each panel.
The asterisk (*) marks the specific bands, while the red rectangle
highlights the blot shown in the main figure.

WB related to Figure 1A

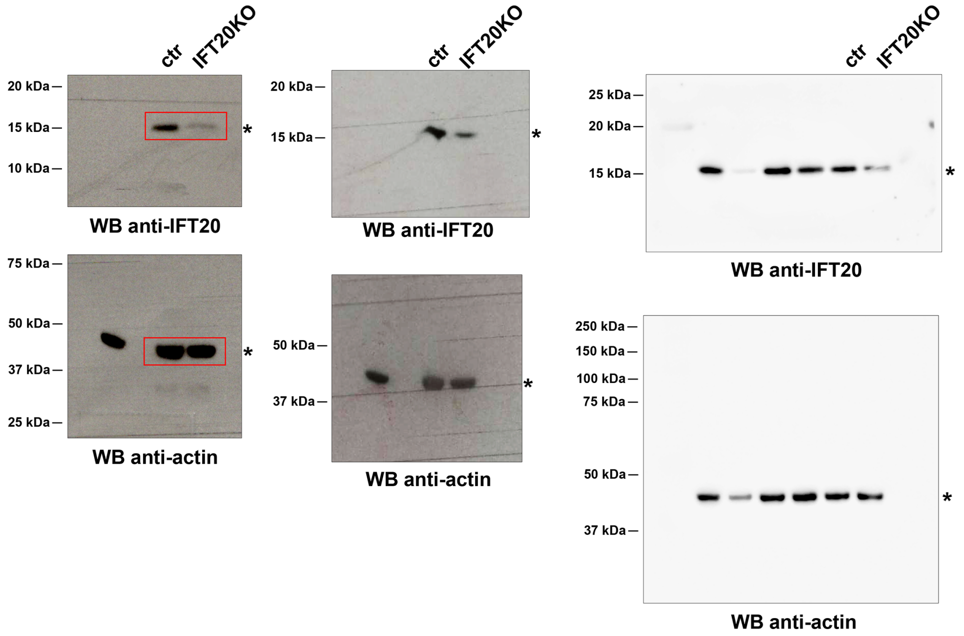

WB related to Figure 1C

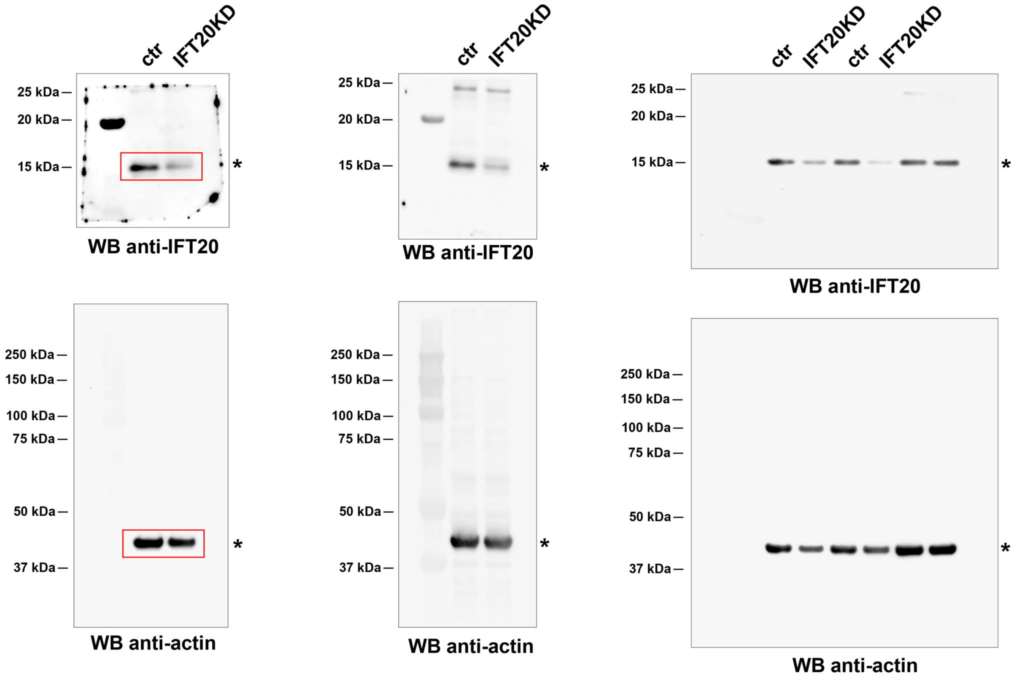

WB related to Figure 3F

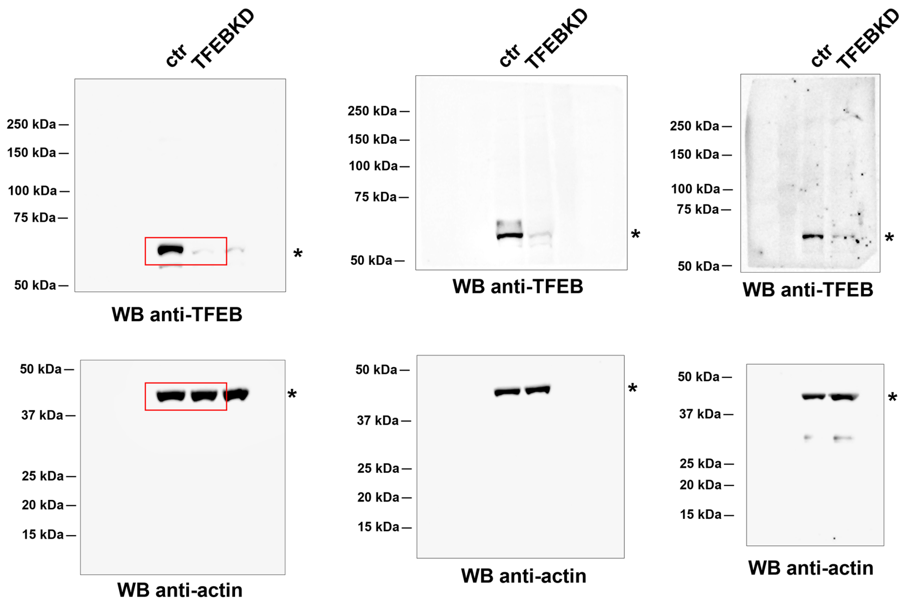

WB related to Figure 4B

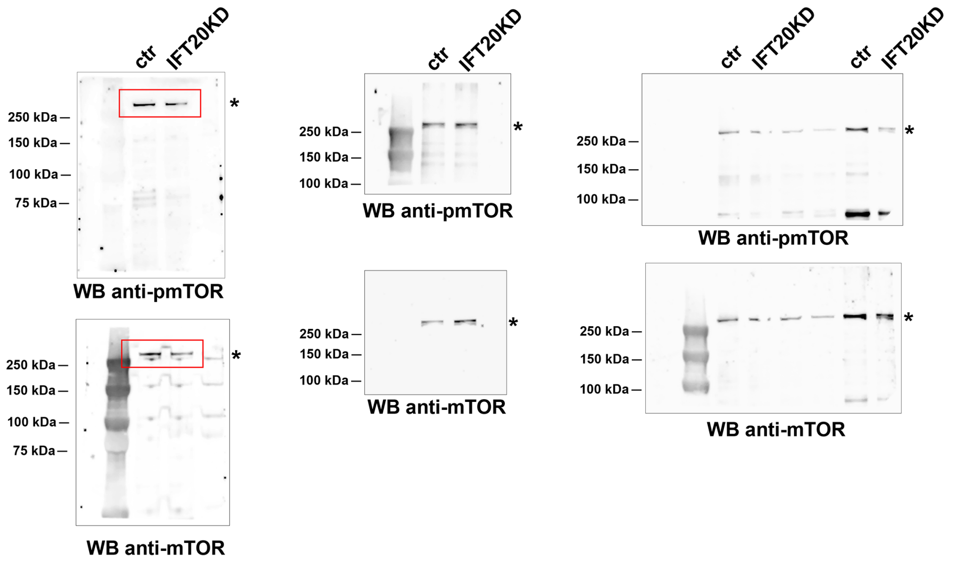

WB related to Figure 4C

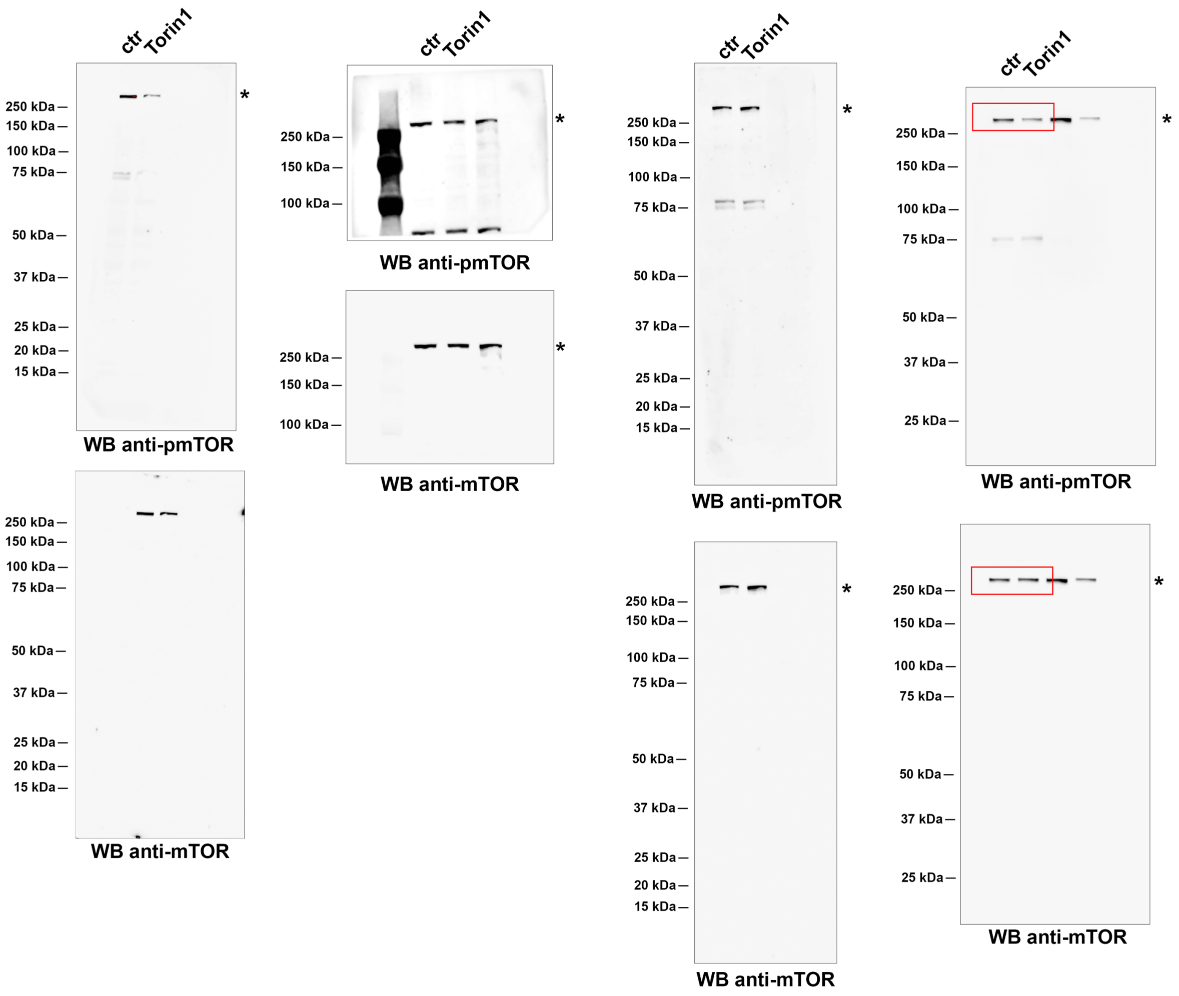

WB related to Figure S2A

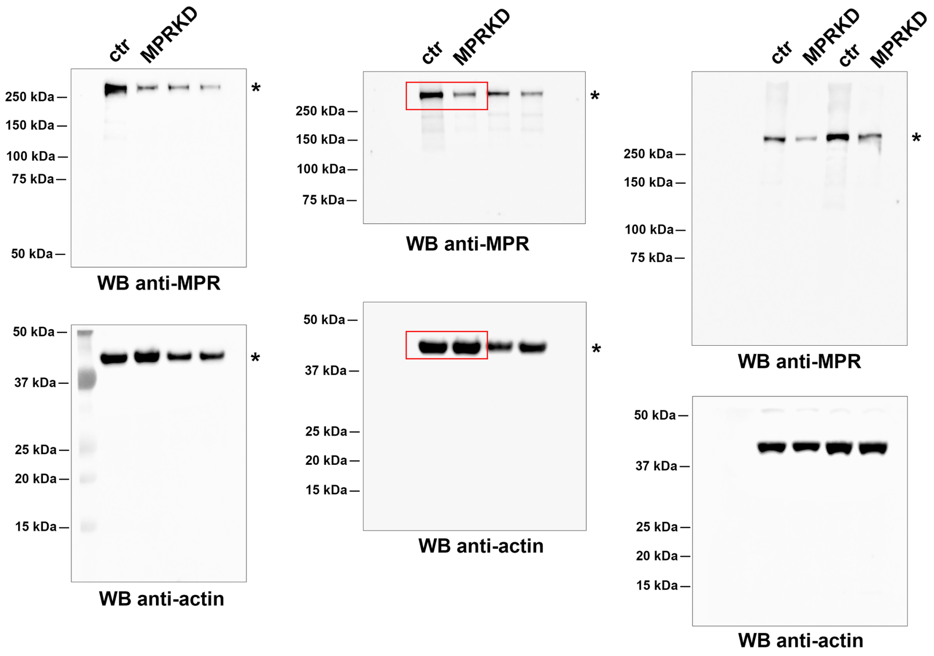

Supplement: Supplementary file 2 — Original data_WB [file 41419_2025_7727_MOESM2_ESM.pdf]
