# Supplementary material for: IFT20 regulates TFEB-dependent lytic granule biogenesis in cytotoxic T lymphocytes by orchestrating the MPR-dependent transport of granzyme B
Source: Cell Death Dis. 2025 May 19;16(1):398. doi: 10.1038/s41419-025-07727-5 (PMC12089405; doi:10.1038/s41419-025-07727-5)
Supplement: Supplementary file 3 — Original data_qPCR [file 41419_2025_7727_MOESM3_ESM.pdf]

Figure 3C

	TFEB					mean	sd
**ctr**	1	1	1	1	1	1	0
**IFT20 KD**	1.43	1.82	1.381	1.503	1.462	1.5192	0.17397615

	LAMP1			mean	sd
**ctr**	1	1	1	1	0
**IFT20 KD**	1.799	1.399	1.487	1.56166667	0.21019356

	CTSD			mean	sd
**ctr**	1	1	1	1	0
**IFT20 KD**	2.36	3.126	2.763	2.74966667	0.38317402

Figure 3D

	GZMA				mean	sd
**ctr**	1	1	1	1	1	0
**IFT20 KD**	1.989	2.068	2.677	2.41	2.286	0.31832373

	GZMB			mean	sd
**ctr**	1	1	1	1	0
**IFT20 KD**	2.21	2.551	2.36	2.37366667	0.17091031

	PRF			mean	sd
**ctr**	1	1	1	1	0
**IFT20 KD**	1.567	1.734	1.966	1.75566667	0.20038047

	SRGN			mean	sd
**ctr**	1	1	1	1	0
**IFT20 KD**	1.409	1.589	1.846	1.61466667	0.21962772

	GNLY			mean	sd
**ctr**	1	1	1	1	0
**IFT20 KD**	2.329	1.775	2.11	2.07133333	0.27901673

Figure 4D

	LAMP1			mean	sd
ctr	1	1	1	1	0
TFEB GFP	11.637	9.72	12.228	11.195	1.31112128

	CTSD			mean	sd
ctr	1	1	1	1	0
TFEB GFP	9.142	10.887	13.248	11.0923333	2.06068686

Figure 4E

	GZMA			mean	sd
ctr	1	1	1	1	0
TFEB GFP	1.549	2.189	1.728	1.822	0.33019237

	GZMB			mean	sd
ctr	1	1	1	1	0
TFEB GFP	2.381	2.119	2.072	2.19066667	0.16650025

	PRF			mean	sd
ctr	1	1	1	1	0
TFEB GFP	4.41	4.398	3.966	4.258	0.25295059

	SRGN			mean	sd
ctr	1	1	1	1	0
TFEB GFP	2.665	2.219	1.984	2.28933333	0.34590509

	GNLY			mean	sd
ctr	1	1	1	1	0
TFEB GFP	2.304	2.549	2.254	2.369	0.15787653

Figure 4G

	LAMP1			mean	sd
ctr	1	1	1	1	0
TFEB KD	0.7115	0.73785	0.85206	0.76713667	0.07471653

	CTSD				mean	sd
ctr	1	1	1	1	1	0
TFEB KD	0.41	0.77503	0.80746	0.6751	0.66416333	0.22070835

Figure 4H

	GZMA				mean	sd
ctr	1	1	1	1	1	0
TFEB KD	0.27	0.72	0.41255	0.80024	0.5506975	0.25087222

	GZMB				mean	sd
ctr	1	1	1	1	1	0
TFEB KD	0.59175	0.76731	0.3831	0.66844	0.60265	0.16305649

	PRF					mean	sd
ctr	1	1	1	1	1	1	0
TFEB KD	0.35995	0.54452	0.77228	0.61729	0.60212	0.579232	0.14876939

	SRGN			mean	sd
ctr	1	1	1	1	0
TFEB KD	0.71554	0.84815	0.76005	0.77458	0.06748847

	GNLY					mean	sd
ctr	1	1	1	1	1	1	0
TFEB KD	0.45607	0.745	0.681	0.69185	0.80296	0.675376	0.13183638

Figure 5E

	GZMA				mean	sd
**ctr**	1	1	1	1	1	0
**Torin 1**	0.772	2.84332	1.99633	2.39761	2.002315	0.89017945

	GZMB				mean	sd
**ctr**	1	1	1	1	1	0
**Torin 1**	0.94306	2.777	2.85968	2.58985	2.2923975	0.9066125

	PRF						mean	sd
**ctr**	1	1	1	1	1	1	1	0
**Torin 1**	1.707	2.843	1.64316	3.09571	2.07578	3.40585	2.46175	0.75196963

	SRGN					mean	sd
**ctr**	1	1	1	1	1	1	0
**Torin 1**	1.52606	1.469	1.74793	1.58349	2.02602	1.6705	0.22440242

	GNLY						mean	sd
**ctr**	1	1	1	1	1	1	1	0
**Torin 1**	1.381	1.529	1.74459	1.35746	1.4325	2.19924	1.60729833	0.32235358

Supplementary figure 2 E

	TFEB				mean	sd
ctr	1	1	1		1	0
MPR KD	1.34434	1.2376	1.23855		1.27349667	0.06135397

	LAMP1				mean	sd
ctr	1	1	1	1	1	0
MPR KD	1.35144	1.39686	1.63582	1.77977	1.5409725	0.20224678

	CTSD				mean	sd
ctr	1	1	1		1	0
MPR KD	1.5541	1.4749	1.27847		1.43582333	0.14190917

Supplementary figure 2 E

	GZMA				mean	sd
ctr	1	1	1	1	1	0
MPR KD	2.55544	1.46537	2.10918	1.52394	1.9134825	0.51735066

	GZMB				mean	sd
ctr	1	1	1	1	1	0
MPR KD	1.9835	1.32076	1.73965	1.40791	1.612955	0.30592842

	PRF				mean	sd
ctr	1	1	1		1	0
MPR KD	2.91082	2.58034	1.8646		2.45192	0.53480169

	SRGN					mean	sd
ctr	1	1	1	1	1	1	0
MPR KD	3.09553	1.72746	1.33478	2.81658	1.33841	2.062552	0.83694715

	GNLY					mean	sd
ctr	1	1	1	1	1	1	0
MPR KD	2.19841	1.54929	2.20427	2.82428	1.88287	2.131824	0.4718319
